# Solid Microneedles for Transdermal Delivery of Amantadine Hydrochloride and Pramipexole Dihydrochloride

**DOI:** 10.3390/pharmaceutics7040379

**Published:** 2015-09-28

**Authors:** Mylien T. Hoang, Kevin B. Ita, Daniel A. Bair

**Affiliations:** 1 College of Pharmacy, Touro University, Mare Island-Vallejo, CA 94592, USA; E-Mail: mylien.hoang@tu.edu; 2 Department of Land, Air, and Water Resources, University of California, Davis, CA 95616, USA; E-Mail: dabair@ucdavis.edu

**Keywords:** transdermal, solid microneedles, microneedle roller, percutaneous flux, burst force

## Abstract

The aim of this project was to study the influence of microneedles on transdermal delivery of amantadine hydrochloride and pramipexole dihydrochloride across porcine ear skin *in vitro*. Microchannel visualization studies were carried out and characterization of the microchannel depth was performed using confocal laser scanning microscopy (CLSM) to demonstrate microchannel formation following microneedle roller application. We also report, for the first time, the use of TA.XT Plus Texture Analyzer to characterize burst force in pig skin for transdermal drug delivery experiments. This is the force required to rupture pig skin. The mean passive flux of amantadine hydrochloride, determined using a developed LC–MS/MS technique, was 22.38 ± 4.73 µg/cm^2^/h, while the mean flux following the use of a stainless steel microneedle roller was 49.04 ± 19.77 µg/cm^2^/h. The mean passive flux of pramipexole dihydrochloride was 134.83 ± 13.66 µg/cm^2^/h, while the flux following the use of a stainless steel microneedle roller was 134.04 ± 0.98 µg/cm^2^/h. For both drugs, the difference in flux values following the use of solid stainless steel microneedle roller was not statistically significantly (*p* > 0.05). Statistical analysis was carried out using the Mann–Whitney Rank sum test.

## 1. Introduction

Parkinson’s Disease (PD) is a disabling, neurodegenerative disorder with no current treatment option to alter the progression of the disease [1,2]. In the United States, PD affects nearly 1.5 million Americans, and according to a recent study, approximately 50,000 new cases are diagnosed each year, and this number is expected to increase substantially as the median age of the population continues to rise [2,3,4,5].

Ultimately, patients suffering from PD exhibit progressive decline in motor function, resulting in significant disability [1,6]. Cardinal symptoms of the disease include resting tremor, bradykinesia, rigidity, and postural instability [7]. Although there is still no cure for PD, various symptomatic therapy options are available in the form of oral medications, surgery, and an FDA-approved rotigotine transdermal patch called Neupro^®^; however, its use focuses on treating idiopathic and early stage PD [1,8]. Available oral therapies include levodopa, dopamine agonists, monoamine oxidase type B inhibitors (MAO-B inhibitors), catechol-*O*-methyltransferase inhibitors (COMT-Inhibitors), amantadine, and anticholinergic agents [1,9,10,11,12].

In a study by the American Academy of Neurology, amantadine, dopamine agonists, and other common antiparkinsonian agents were examined. This study found that amantadine hydrochloride relieved PD symptoms by acting on certain postsynaptic receptors in the striatum, whereas dopamine agonists bind to and activate dopamine receptors [7,12]. In the literature, amantadine has shown to be effective and is mostly utilized to treat peak-dose levodopa-induced dyskinesia (LID) in patients with advanced stage PD [13].

It has been suggested that non-ergot dopamine agonists like pramipexole dihydrochloride may reduce the duration of “off-time” episodes [12].

Chase and Oh have hypothesized that nonphysiologic stimulation of striatal dopaminergic receptors may trigger adaptive responses in the basal ganglia, which contribute to the appearance of parkinsonian symptoms and later to the dyskinesias [14]. Our rationale in developing transdermal formulations for pramipexole is that a steady zero-order drug input is capable of reducing peak-trough variations and this leads to continuous stimulation of dopamine receptors [15]. This models normal physiological conditions in healthy individuals and from a therapeutic perspective less complications [15]. Transdermal drug delivery will lead to a more stable plasma concentration, reduced side effects and better patient compliance. Our long term goal is the development of a transdermal system for the delivery of pramipexole and amantadine.

It is important to expand the scope of transdermal drug delivery options as an alternative form of administration. Transdermal drug delivery has expanded greatly in the last decade as an exciting potential therapy approach that provides considerable benefits. For instance, this route of administration is capable of providing drug delivery at a controlled and constant rate while also being non-invasive and simple to use, which would be advantageous for patients that suffer from advanced stage PD.

Microneedle-facilitated transdermal delivery has the potential of increasing skin permeability for many compounds that normally do not penetrate the stratum corneum (SC), which is the outermost layer of the skin and the major barrier for transdermal drug delivery [16,17,18]. As an alternative delivery route, microneedle-assisted transdermal drug delivery is minimally invasive and capable of providing sustained drug release, thus reducing dosing frequency. When administered to the skin, these micron-sized needles do not reach the nerve fibers located within the dermis layer of the skin, making this approach a painless route of administration [19]. Additionally, a microneedle delivery system can be self-administered, and the ease and convenience of this application system could potentially improve patient adherence to medication therapy, which is especially important for patients suffering from advanced-stage PD, since improving quality of life is of great significance [20].

Pramipexole is a cationic drug with a net positive charge at physiological pH (pKa 5.0 and 9.6) and an octanolphosphate buffer (pH 7.4) partition coefficient of 0.135 [21]. It has a molecular weight of 284.25 g/mol [22] and a pharmacokinetic half-life of 6–8 h [23]. A transdermal delivery system for pramipexole might provide benefits compared to oral dosage forms. Apart from the pharmacokinetic aspects, adherence to therapy is an issue for elderly patients including those that suffer from PD. Some elderly patients simply forget to take their medications, especially those on multiple medications.

Amantadine has a molecular weight of 187.71 g/mol and a partition coefficient of 2.44 [24]. The pharmacokinetic half-life of the drug is 10 h [25]. Patience compliance will be greatly improved if amantadine is formulated into a transdermal patch.

The aim of this study was to investigate the influence of microneedles on transdermal delivery of amantadine hydrochloride and pramipexole dihydrochloride across porcine ear skin *in vitro*. Stainless steel microneedle rollers were utilized to create microchannels in the SC to assist with the delivery of amantadine hydrochloride and pramipexole dihydrochloride across porcine skin.

## 2. Materials and Methods

### 2.1. Materials

Stainless steel microneedle rollers (500 μm) were purchased from Pearl Enterprises LLC. (Lakewood, NJ, USA) Amantadine hydrochloride, pramipexole dihydrochloride, and 0.1 M isotonic phosphate buffered saline (PBS) were purchased from Sigma–Aldrich Co. (St. Louis, MO, USA). Amantadine hydrochloride and pramipexole dihydrochloride were reconstituted using PBS. The water was processed using NanoPure Infinity Ultrapure water purification system (Barnstead, Dubuque, IA, USA).

### 2.2. Methods

#### 2.2.1. Skin Preparation

Experiments were approved by the Institutional Animal Care and Use Committee (IACUC) and Institutional Biosafety Committee (IBC) of Touro University, Mare Island-Vallejo, CA, USA. Frozen porcine ears were obtained from Animal Technologies, Tyler, TX, USA. Skin pieces were thawed at ambient temperatures and carefully shaved using an electric clipper (Wahl, Sterling, IL, USA). Full thickness skin was prepared by removing the subcutaneous fat from the underlying cartilage. The average thickness of the skin membrane was measured with a Digimatic Micrometer (Mitutoyo, Tokyo, Japan). Skin samples were maintained at −20 °C for storage. Prior to experiments, the skin samples were thawed at room temperature before use.

#### 2.2.2. *In Vitro* Diffusion Studies

*In vitro* permeation experiments were conducted using a six-celled, static, vertical Franz diffusion cell system (PermeGear, Hellertown, PA, USA). Each cell contained a top donor compartment and a lower receptor compartment with a magnetic stirrer, sampling port, and water jacket maintained at 37 °C to simulate normal body temperature by Thermo Haake DC10-P5/U heating circulator bath (ThermoFisher Scientific, Waltham, MA, USA). The receptor compartment diffusion area was 1.77 cm^2^ with a volume capacity of 12 mL filled with PBS. Skin samples obtained from different pigs were placed between the upper donor chamber and lower receiver chamber of the vertical Franz diffusion cells, which were sealed using high-vacuum grease (Dow Corning, Midland, MA, USA) and a metal clamp. Solid microneedle rollers were used to created microchannels in porcine skin. Full thickness microneedle-treated porcine skins were mounted on three-receptor compartments and untreated porcine skins were placed on the remaining three-receptor compartments to serve as controls, and diffusion experiments for the two drugs were replicated six times (*n* = 6). The microneedle roller was applied fifteen times to each skin sample to increase the number of microconduits for enhanced transdermal drug delivery. Before each microneedle application, the skin samples were rotated 90° and the microneedles were applied with a force of 9.07 kg for ~1 min per application. Each experiment was performed using 1 mL of either amantadine (~50 mg/mL) or pramipexole (~1 mg/mL), which was loaded onto the skin samples via the donor compartment and covered with parafilm and aluminum foil to reduce evaporation. The sampling ports were also covered with parafilm to further reduce evaporation. Aliquots of 1 mL were removed from the sampling port at the interval of 2 h for a 12-h period and placed into vials for high performance liquid chromatography-mass spectrometry (HPLC–MS). Receptor chambers were replenished with an equal volume of fresh, pre-warmed PBS maintained at 37 °C. All samples were stored at 4 °C until analysis by LC–MS. The cumulative amount of each drug permeating the excised full thickness porcine skin was plotted as a function of time. The slope and intercept of the linear portion were derived by linear regression analysis. Steady state flux was calculated from the linear portion of the cumulative amount versus time curve. The cumulative amount of each drug permeated (*Q*_s_, μg/cm^2^) for 12 h was also calculated.

#### 2.2.3. High-Performance Liquid Chromatography–Mass Spectrometry Analysis (HPLC–MS)

HPLC–MS analysis was performed using an Agilent series 1200 HPLC with diode-array and Agilent 6320 Ion Trap mass spectrometer detectors (Agilent Technologies, Palo Alto, CA, USA). Chromatographic separation was carried out on the reverse-phase Agilent Zorbax Eclipse Plus C18 (100 mm × 2.1 mm, 3.5 microns) analytical column, which was protected by a guard column with the same stationary phase (12.5 mm × 4.6 mm, 5 microns) (Agilent Technologies, Palo Alto, CA, USA). The column temperature was set at 40 °C, and the autosampler temperature was set at 4 °C. The mobile phase consisted of 0.1% formic acid in water (solvent A), and 0.1% formic acid in methanol (solvent B). The solvent gradient was performed at 0.4 mL/min with an initial condition of 5% of mobile phase B. Mobile phase B was increased to 95% at 2 min and held at 95% B until 6 min at which Mobile phase B was then reduced to 5% at 7 min. A post-run time of 2 min for mobile phase equilibration was used after each sample run. Calibration curve standards were freshly prepared in PBS buffer solution. The MS data were collected in positive electro spray ionization tandem mass spectrometry (ESI MS/MS) mode. Nebulizer temperature was 350 °C, nebulizer pressure was 50 psi, and the drying gas flow rate was 10.0 L/min. Compounds were quantified in positive ESI MS/MS mode by quantifying the specific product ion. A UV spectrum was only collected for pramipexole dihydrochloride at the wavelength of 263 nm because the structure of amantadine hydrochloride is not conjugated; therefore, mass spectrometric detection was used. For amantadine hydrochloride, ion transitions *m*/*z* was 152.0→135.1 and for pramipexole dihydrochloride, ion transitions *m*/*z* was 212→153.

#### 2.2.4. Visualization of Microchannels

Microchannel imaging studies were conducted at the Comparative Pathology Lab of the University of California, Davis. Porcine skin samples were treated with a 500 μm long microneedle roller for ~1 min and then stained with a margin marking dye (American MasterTech Scientific Inc., Lodi, CA, USA). Following treatment, skin samples were immediately rinsed with saline to remove excessive dye. As the control, untreated porcine skin samples were similarly stained with margin marking dye for ~1 min and immediately rinsed with saline. Pictures of the treated and untreated skin samples were collected using a digital camera (Canon, Melville, NY, USA).

#### 2.2.5. Characterization of Microchannel Depth by Confocal Laser Scanning Microscopy (CLSM)

CLSM was performed at the Cellular and Molecular Imaging Lab of the University of California, Davis to characterize the depth of the created microchannels. Excised full thickness porcine skin was treated with a microneedle roller device. The porated site was treated with 200 μL of a fluorescent dye, Alexafluor 488 (Life Technologies, Eugene, OR, USA) for ~1 min, after which the site was blotted with kimwipes to remove excess dye. Samples of microneedle treated porcine skin were mounted onto Tissue-Tek Cryomold (Sakura Finetek Inc., Torrance, CA, USA) and covered with OCT embedding media (Sakura Finetek Inc., Torrance, CA, USA) before freezing in dry ice storage at −80 °C. Skin samples were maintained on dry ice before cry-sectioning using a Leica CM1950 Cryostat (Leica Biosystems, Buffalo Grove, IL, USA). Samples were cry-sectioned to 10 μm-thick vertical sections and mounted onto glass slides. Transmission images of the skin samples were examined and recorded using a Leica TCS LSI laser scanning confocal microscope at 5× magnification. Excitation was carried out at 488 nm and emission at 520 nm. X-Z sectioning was performed to detect depth of dye fluorescent penetration. The frame size was set to 1024 × 1024 pixels, and gain and offset were maximized to enhance contrast of images. Depth of the microchannels was estimated indirectly based on migration of the Alexafluor 488 down the microchannel to indicate dye permeation.

#### 2.2.6. Burst Strength

Burst strength of skin was evaluated using a TA.XT Plus Texture Analyzer (Texture Technologies, Hamilton, MA, USA) to measure the force required to rupture the skin. The burst strength study was carried out using a burst rig which uses a 5 mm spherical stainless steel ball probe attached to a probe adapter connected to the load cell (50 kg maximum load).

#### 2.2.7. Data Analysis

Flux values were calculated using the steady-state portion of the cumulative amount *versus* time curves. Six replicates were used for the study. Drug concentration was corrected for sampling effects according to Equation 1, proposed by Hayton *et al* and used by other investigators [26,27,28].

(1) $$C_{n}^{1}=C_{n}\left(\frac{V_{T}}{V_{T}-V_{S}}\right)\left(\frac{C_{n-1}^{1}}{C_{n-1}}\right)$$

In this equation, $C_{n}^{1}\;$is the corrected concentration and $C_{n}$ represents the measured concentration in the *n*th sample. $V_{T}$ is the total volume of the receiver fluid (12 mL) and $V_{S}$ represents the volume of sample withdrawn from the receiver fluid (1 mL). While $C_{n-1}^{1}$ and $C_{n-1}$ are corrected and measured concentration, respectively in (*n* − 1)th sample.

#### 2.2.8. Statistical Analysis

Statistical analysis was performed using Sigmastat (Systat Software, San Jose, CA, USA). Mann–Whitney Rank sum test was carried out to determine statistical significance. Mean of replicate measurements (*n* = 6) with corresponding standard deviation was used to plot the graphs.

## 3. Results

### 3.1. Characterization of Microneedle Array and Microneedle Roller

**Figure 1:**
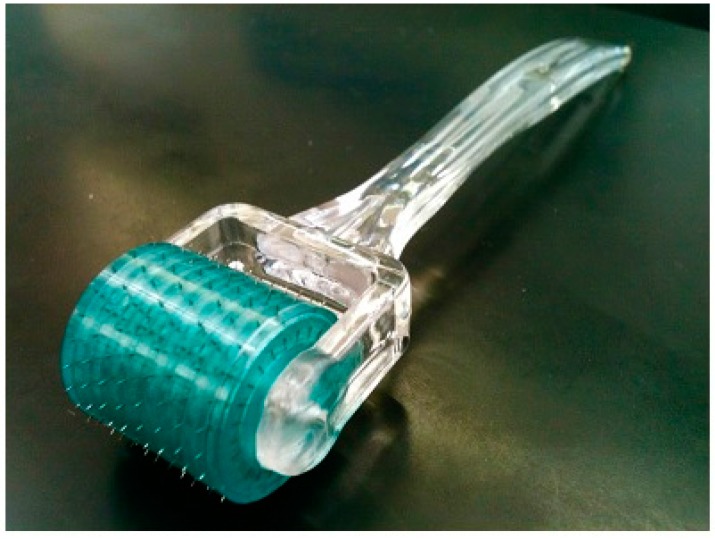
Stainless steel microneedle roller, density of 192 microneedles and 500 μm in length per microneedle.

**Figure 2:**
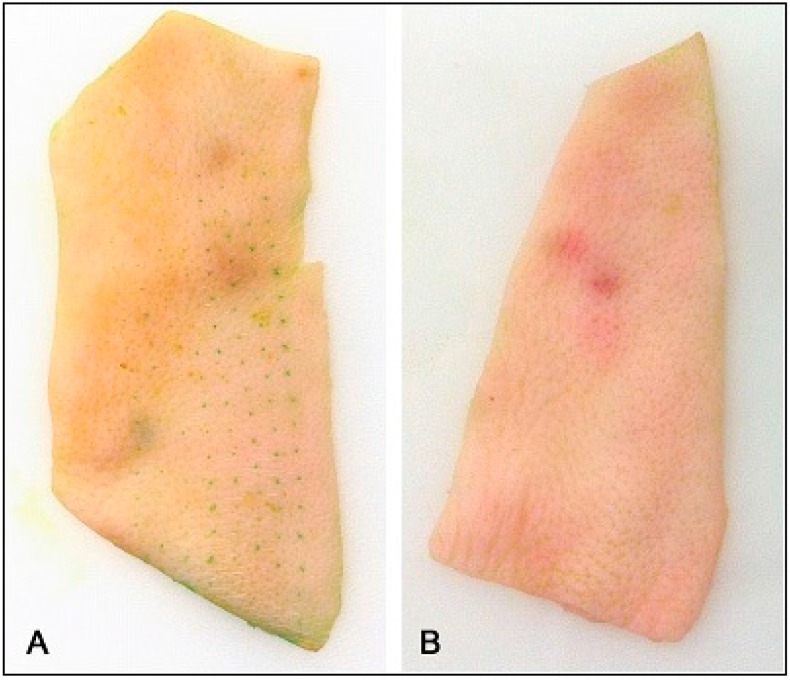
Microchannel visualization using margin marking dye. (**A**) Porcine skin treated with microneedle roller. (**B**) Non-treated porcine skin as the control.

**Figure 3:**
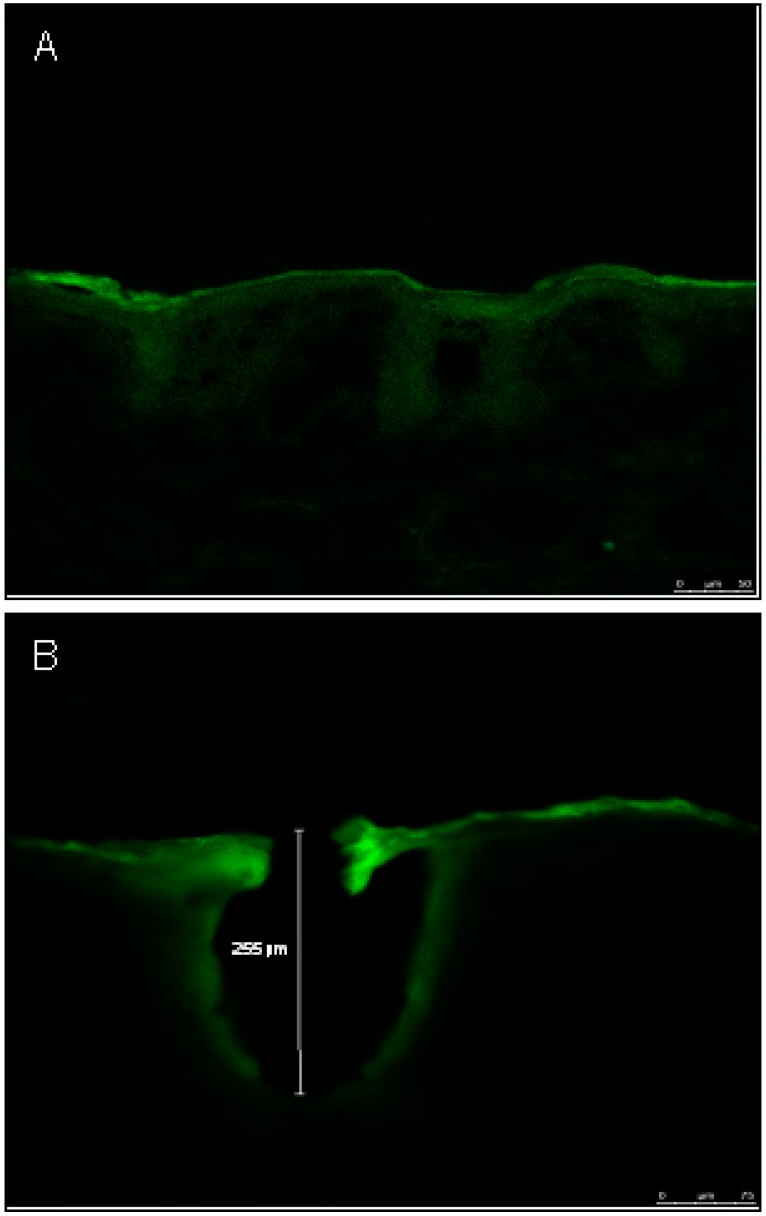
Representative depth of a single microchannel by confocal laser scanning microscopy (CLSM). (**A**) Untreated porcine skin. (**B**) Microneedle treated porcine skin showing a microchannel depth of 255 μm.

When microneedles are applied to the surface of the skin they disrupt the barrier of the SC and create microchannels, resulting in increased drug penetration [16,29]. A stainless steel microneedle roller was used in this study to deliver the antiparkinsonian agents amantadine hydrochloride and pramipexole dihydrochloride across porcine skin. The microneedle roller contains microneedles protruding from a cylindrical surface measuring 500 μm in length per microneedle with a density of 192 needles (Figure 1). Following microneedles application, microchannels were visualized, as shown in Figure 2A, and the microchannel depth was characterized, as shown in Figure 3B.

### 3.2. Visualization of Microchannels

After application of the microneedle roller across the skin surface, the created microchannels can be visualized. Figure 2A is a picture of full thickness porcine skin stained with margin marking dye following treatment with one pass of the microneedle roller. The areas of the skin that were disrupted by the microneedles took up the dye identifying the created microchannels immediately after application of the solid microneedle roller. The portion of the skin surrounding the microchannels maintained normal structure and intact stratum corneum. Figure 2A shows the pattern of the created microchannels aligned symmetrically, mirroring the pattern of the microneedle roller. A control was employed to demonstrate that the microchannels on the surface of the skin are due to the microneedles alone (Figure 2B).

### 3.3. Characterization of Microchannel Depth by Confocal Laser Scanning Microscopy (CLSM)

Porcine skin samples were imaged and recorded to characterize the depth of the microchannel created by the microneedle roller from the surface of the skin using CLSM (Figure 3A,B). The skin samples were treated with Alexafluor 488 and the binding of the Alexafluor fluorescent particle along the microchannels indicates the depth of the created microchannel, which resulted in a mean depth of 319.50 ± 71.05 μm (*n* = 4). Figure 3 is a representative image of a single microchannel displaying the microchannel depth of 255 μm. In contrast, the non-disrupted skin sample served as the control, and results revealed no created microchannel (Figure 3A). It is important to note that microchannel depth is directly influenced by various factors such as the elasticity of the skin, application force [30].

### 3.4. Burst Strength

The skin has both elastic and viscous properties and must be strong and ductile to provide protection to the body and prevent foreign substances from entering through the skin. In this study, for the first time, a Texture Analyzer was used to test the skin to measure the burst force from porcine skin samples. Burst force strength indicates that the skin is flexible and, at the same time, rigid enough to prevent the deformation from foreign objects and agents that are imposed upon the stratum corneum (SC) surface. Table 1 shows that the results of burst strength and distance to burst porcine ear skin, which indicates that the skin samples are slightly elastic and rupture with an average force of 187.5 N at a distance of 7.76 mm.

**Table 1:** Burst force required to rupture porcine skin (*n* = 6).

Initial Gradient (N/s)	Mid Gradient (N/s)	Final Gradient (N/s)	Work to Burst Skin (N/s)	Burst Force (N)	Distance to Burst (mm)
13.75 ± 2.11	43.98 ± 8.17	62.14 ± 11.54	341.24 ± 42.44	187.54 ± 19.38	7.77 ± 0.63

### 3.5. In Vitro Diffusion Studies

**Figure 4:**
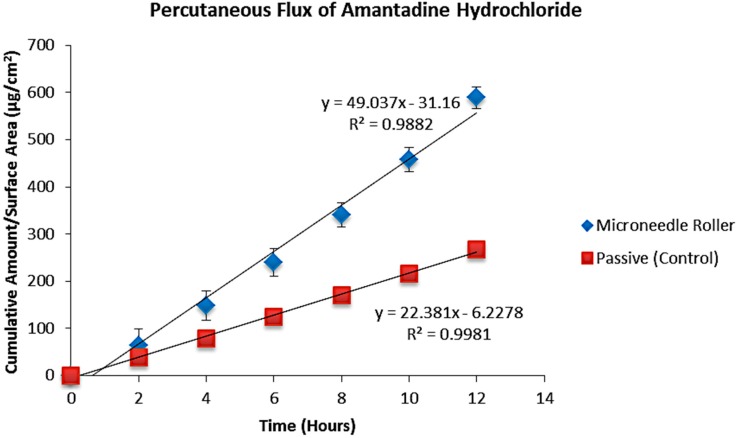
*In vitro* transdermal permeation of passive and microneedle-facilitated amantadine hydrochloride after microneedle roller application across porcine ear skin (500 μm length, 540 needles per square centimeter density).

**Figure 5:**
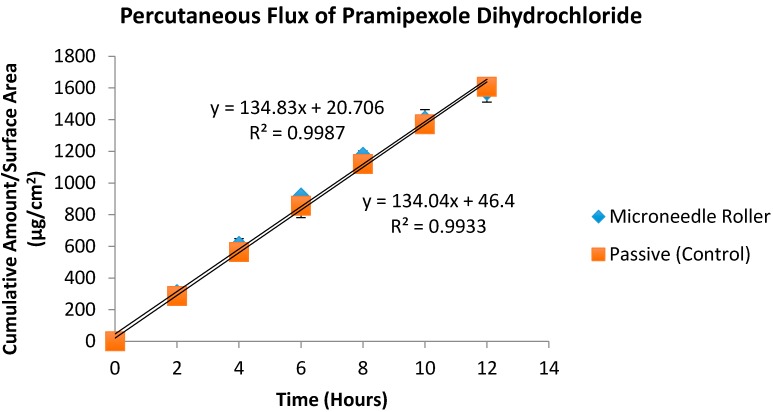
*In vitro* transdermal permeation of passive and microneedle-facilitated pramipexole dihydrochloride after microneedle roller application across porcine ear skin (500 μm length, 540 per square centimeter density).

An *in vitro* diffusion study was carried out to identify the influence of solid microneedles on the transcutaneous absorption of amantadine hydrochloride and pramipexole dihydrochloride. This study was performed using porcine ear skin as a representative model of human epidermal membrane [31,32]. In our experiments, the mean porcine skin thickness was 761 ± 0.18 µm. LC–MS was used to determine the concentration of the drug in the samples and flux values were determined for both agents using the slope of the steady-state portion of the cumulative amount versus time curves and concentration was corrected for sampling effects according to Equation 1. Table 2 shows a comparison of the final cumulative amounts of both drugs after 12 h (*Q*_s_) following microneedle treatment to passive permeation. A microneedle roller was applied to full thickness pig ear skin at the beginning of experiments, and the amounts of amantadine and pramipexole delivered after 12 h are shown in Table 2 (589.26 and 1583.43 µg/cm^2^ respectively). Table 3 shows a comparison of transdermal flux for both drugs. The passive transdermal flux for amantadine hydrochloride was 22.38 ± 4.73 µg/cm^2^/h, while microneedle-facilitated flux was 49.04 ± 19.77 µg/cm^2^/h (Figure 4). For pramipexole, passive transdermal flux was 134.83 ± 13.66 µg/cm^2^/h, while microneedle-facilitated flux was 134.04 ± 0.98 µg/cm^2^/h. The percutaneous flux of each drug was plotted as a function of time (Figure 5).

**Table 2:** Cumulative amount after 12 h (*Q*_s_, μg/cm^2^ ± SD) of amantadine and pramipexole following treatment with a 500 μm long microneedle roller. Passive flux values served as controls (*n* = 6).

**Amantadine *Q*_s_**	**Passive (Control) *Q*_s_ (μg/cm^2^)**	**Microneedle *Q*_s_ (μg/cm^2^)**
Mean	267.65 ± 14.07	589.26 ± 23.13
**Pramipexole *Q*_s_**	**Passive (Control) *Q*_s_ (μg/cm^2^)**	**Microneedle *Q*_s_ (μg/cm^2^)**
Mean	1607.86 ± 35.77	1583.43 ± 72.99

**Table 3:** Transdermal flux (μg/cm^2^/h ± SD) of amantadine and pramipexole following treatment with a 500 μm long microneedle roller. Passive flux values served as controls (*n* = 6).

**Amantadine Flux**	**Passive (Control) Flux (μg/cm^2^)**	**Microneedle Flux (μg/cm^2^)**
Mean	22.38 ± 4.73	49.04 ± 19.77
**Pramipexole Flux**	**Passive (Control) Flux (μg/cm^2^)**	**Microneedle Flux (μg/cm^2^)**
Mean	134.83 ± 13.66	134.04 ± 0.98

## 4. Discussion

The skin is a composite material consisting of a collagen-rich fibrous network embedded in a proteoglycan-rich matrix [33]. The main fibrous constituents, collagen and elastin, provide structural stiffness and elasticity to the skin while the ground matrix is responsible for its viscous nature at low loads [33]. Effectiveness of microneedle-mediated transdermal drug delivery depends on a number of factors including the mechanical properties of the skin. The inherent elasticity and irregular surface of the skin is a challenge for reproducibility of microneedle flux values [34]. In this paper we seek, for the first time, to introduce burst strength as a parameter that can be used to characterize pig skin in transdermal drug delivery experiments. Burst strength is the pressure at which pig skin will burst. This parameter measures resistance to rupture and is a function of tensile strength as well as extensibility of the skin. It is thus a composite strength property that is affected by other properties of the skin, principally tensile strength and stretch. It can be determined by using a TAXT-analyzer and is expressed in Newtons (N).

The focus of our study was on transdermal delivery of therapeutic agents used in the management of PD. The underlying pathology of PD is due to the gradual loss of dopaminergic neurons in the substantia nigra pars compacta of the basal ganglia [35]. Several studies speculate that genetics, environmental toxins, and several pathological processes such as oxidative stress, apoptosis and mitochondrial DNA defects might be involved in the pathway leading to the degeneration of dopamine neurons; however, there is no definitive proof that any one of these is critically involved, which leaves the cause of PD largely unknown [4,36].

Levodopa is the most potent oral medication for the symptomatic treatment of PD [1,37,38,39]. However, levodopa is not commonly used as initial therapy in the treatment of PD due to its side effects. This phenomenon was first reported by Cotzias *et al.*, followed by subsequent studies showing that long-term treatment with levodopa becomes less effective in eliminating motor symptoms and produced levodopa-induced dyskinesia (LID), a hyperkinetic side effect of levodopa therapy in PD patients [13,40,41,42,43]. Furthermore, later studies have also shown that early use of levodopa may predispose patients to develop long-term motor complications, such as dyskinesia, dystonia, and these symptoms may prematurely return or worsen before the next dose is due, which is called “wearing off” [13,44,45]. These complications has been reported especially in young-onset PD patients [46].

This growing recognition of the complexity involved in long-term management and pulsatile stimulation of dopamine receptors that lead to motor fluctuations that often develop and compromise the effectiveness of long-term levodopa administration in PD patients emphasizes the need for improved therapy and potential alternative drug delivery options [45]. However, developing a drug to be delivered across the skin for the treatment of advanced-stage PD is challenging.

Successful transdermal absorption requires a drug to cross the stratum corneum barrier (SC), which is the outermost layer of the epidermis, and ultimately enter the systemic circulation [47,48,49]. The SC contains a unique structure comprised of a lipid-rich matrix embedded with corneocytes, which are dead, partially desiccated, and keratinized epidermal cells [17]. Although the SC layer is only about 10–15 μm thick, and only accounts for 0.1 mm of the skin’s 1.5 mm thickness, this unique layer is the major barrier against drug permeation [17,48,50]. The structure of the SC can be explained in terms of the “brick and mortar” model, in which the corneocytes represent the bricks, and the intercellular lipid matrix acts as the mortar [19,51]. Due to the limiting parameters of the SC, there are currently only about 40 transdermal products on the market containing 19 active ingredients [17,19].

Amantadine is used in the treatment of tremors as well as dyskinesia, but its mechanism of action is not clearly elucidated [7,11,15,16]. Several literature sources report that amantadine is a weak noncompetitive *N*-methyl-d-aspartate (NMDA) glutamate receptor antagonist and suggest that amantadine acts by blocking NMDA glutamate and acetylcholine receptors thereby promoting the release of dopamine, while others suggest that the mechanism is unknown [3,17]. Regardless of the mechanism of action, amantadine has been shown to be effective and is mostly utilized to treat peak-dose LID in patients with advanced stage PD [13]. On the other hand, pramipexole dihydrochloride is a dopamine agonist and is effective in reducing the duration of “off-time” episodes [12,38,39].

Ghosh and co-authors report that normal healing processes of the skin result in the resealing of micropores formed after application of microneedles within 48–72 h under occlusion. Therefore, a drug can be delivered across microneedle-treated skin for 3 days under occlusion [52]. Although we did not investigate the size of microchannels in our study, there is a report in the literature which showed that the mean diameter of microconduits created by a microneedle roller at the surface of the skin was approximately 70 μm [30]. In that project, the authors combined confocal microscopy with micron-sized fluorescent particles.

Based on our results, the stainless steel microneedle roller was capable of creating microchannels in the SC as demonstrated from the microchannel visualization studies and confocal images, but there was no statistically significant increase in percutaneous absorption of amantadine hydrochloride or pramipexole dihydrochloride across porcine skin. Although there was a 1.57-fold increase in mean flux for amantadine hydrochloride, the difference in flux following the use of solid stainless steel microneedle roller was not statistically significant (*p* < 0.05). The difference between passive and microneedle-facilitated fluxes of pramipexole was also not statistically significant (*p* < 0.05).

Several factors can cause lack of transdermal flux enhancement, which we witnessed in this project. The physiochemical properties of a drug can influence the rate of transdermal drug penetration. The physiochemical properties of a drug include: molecular weight, diffusion coefficient, drug concentration, melting point, pH, and charge [17,53,54].

For a drug molecule to passively diffuse across the SC, it must have a low molecular weight, typically less than 500 Daltons. Additionally, since inside the skin is an aqueous environment and the skin is made of a lipid-rich bilayer, it is important that the drug molecule possess both lipid and aqueous solubility that is suitable for percutaneous absorption. Thus, the ideal drug compound would possess an aqueous solubility of greater than 1 mg/mL and lipophilicity between 1–3 [17,55].

However, these properties would likely not have influenced percutaneous absorption of our compounds of interest, as both substances are relatively small compounds with appropriate water solubility and lipophilicity that are theoretically suitable for passive diffusion across the SC. As mentioned, the molecular weights, water solubility, and lipophilicity of amantadine hydrochloride and pramipexole dihydrochloride are 187.71 Da, 50 mg/mL, with a log*P* of 2.4 and 284.25 Da, >20 mg/mL, with a log*P* of 1.2 respectively.

Low diffusion coefficient is a possible explanation for the low flux values observed in this study. Since passive drug absorption through the skin is governed by diffusion, the drug molecule of interest must move according to the concentration gradient from high to low [55]. Commercially, amantadine hydrochloride is available as 100 mg oral capsule or tablet often prescribed at twice a day as monotherapy and may increase to 400 mg/day in divided doses, while pramipexole dihydrochloride is available as immediate release in the dosage range of 0.125, 0.25, 0.5, 0.75, 1, 1.5 mg and extended release oral tablet in the dosage range of 0.375, 0.75, 1.5, 2.25, 3, 3.75, 4.5 mg. In the present study, 50 mg/mL for amantadine hydrochloride and 1 mg/mL for pramipexole dihydrochloride was used.

While most studies using microneedles for transdermal drug delivery have reported flux enhancement [56,57], there are some studies that have reported lack of flux enhancement [58]. A recent study by Vitorino *et al.* did not observe significant transdermal flux enhancement after application of microneedles. Their results showed that the application of a microneedle device as pretreatment led only to a slight but not statistically significant increase in the transdermal permeation rate of olanzapine and simvastatin. Their findings suggested that the use of a nanostructured lipid carrier might have a greater impact on skin permeation than the active enhancement strategy of microneedle application [27].

Furthermore, it may be possible that the low diffusion coefficient of the drugs may have led to the lower flux values. Equation 2 shows that there is a relationship between surface area, membrane thickness, partition coefficient, and diffusion coefficient [59,60].

(2) $$J_{ss}=\;\frac{ADKC_{V}}{h}$$

This equation is derived from Fick’s first law of diffusion [59] and describes steady state flux across membranes. The equation describes the rate of drug flux *J*_ss_ of the diffusing agent through unit area *A* of the membrane as being proportional to the velocity of molecular movement though the diffusional medium or diffusion coefficient *D* and to the concentration gradient measured across the membrane. In this equation *D* is the effective diffusional pathway of the membrane and the vehicle-membrane partition coefficient *K*, which may be further defined as the ratio between the concentration of the permeant in the membrane at the donor-membrane interface and the vehicle in which it is applied (*C*_V_). According to Lane, this equation indicates that increased flux should be achieved by a change in diffusion coefficient *D*, partition coefficient *K*, and applied drug concentration *C*_V_. Therefore, the low diffusion coefficient of the drugs we investigated in this study may have been responsible for the lack of flux enhancement with microneedles [60].

A further possible explanation for the low flux values may have been the so-called “bed of nails” effect. Studies such as Yan *et al.* and Badran *et al.* evaluated the effect of microneedle length and density on transdermal drug delivery [16,61]. Yan *et al.* observed significant enhancement in acyclovir flux across human epidermal membrane pretreated with microneedle arrays of 400 μm needle length and 2000 needles/cm^2^ in needle density, but a lower enhancement of drug flux was observed for the microneedles with same needle length but a with a higher needle density of 5625 needle/cm^2^ [61]. Although the applied force on the microneedles was the same per array, this study found that the force to an individual needle would be smaller for microneedle arrays with higher needle density. This phenomenon is similar to the “bed of nails” effect, in which there are a sufficient number of sharp nails on a bed such that the weight distributed among the nails is not sufficient to exert the pressure needed by each nail to break the skin. Therefore, a lower-density microneedle array would produce a more effective puncturing of the skin. It is possible that the application method of applying a constant force on all the microneedle arrays could benefit the low-density microneedles to some extent because there was more force applied to each single needle for the low-density microneedles.

A similar trend was observed by Badran *et al.*; this study focused on the use of microneedle rollers (Dermaroller^®^) of various lengths ranging from 150, 500, and 1500 μm on drug permeation [16]. This study found that the use of the medium and shortest microneedle rollers enhanced drug delivery. Furthermore, the shortest microneedle roller led to the highest drug deposition in the SC while the longer microneedle models deposited drugs in the SC in similar (500 μm) or even lower (150 μm) amounts when analyzed after incubation of the skin. To our knowledge, this is the first time that the microneedle approach has been proposed for percutaneous transport of amantadine hydrochloride and pramipexole dihydrochloride. Though several reports exist in the literature regarding the use of microneedles for transdermal drug delivery, there are still significant challenges. As seen in this publication and others (Vitorino *et al.*) the use of enhancement techniques does not always result in percutaneous flux increases, and so it is neither trivial nor routine to test the effect of microneedles on transdermal delivery of each drug.

In summary, the results of our study using a stainless steel microneedle roller indicate that the microneedle roller device can be used to create microchannels in the SC and is theoretically capable of enhancing *in vitro* transport of our compounds of interest across the skin. Our ultimate goal is to increase the flux values for these drugs, and combined techniques with sonophoresis and chemical penetration enhancers will be explored. It is important to keep in mind that transcutaneous flux is a complex phenomenon that depends on several factors such as microneedle geometry and the physiochemical properties of the compound [62].

## 5. Conclusions

Application of a microneedle roller did not lead to a statistically significant increase in transdermal flux values for amantadine and pramipexole in this study. The reason may be insufficient increase in diffusion coefficient. Our future studies will examine combined techniques such as microneedles in conjunction with sonophoresis or chemical permeation enhancers as a means of increasing transdermal flux.

## Glossary

Burst force, the peak force which the probe detected as it moved into the sample; Distance to burst, the distance from the start of the probe touching the sample to the peak force the probe detected (where the probe penetrated the tissue); Work to burst, the area under curve from the start of the probe touching the sample to the peak force; Initial gradient (N/s), the slope of the line from 1% of maximum force to 20% of maximum force; Mid gradient, slope of the line from 40% to 60% of maximum force; Final gradient, slope of the line from 80% to 99% of maximum force.
